# CT Colonography: A Guide for Clinical Practice

**DOI:** 10.1002/jmrs.38

**Published:** 2014-01-06

**Authors:** Frank Parrish

**Affiliations:** I-TeleRADBuilding 2 level 3, 540 Springvale rd, Glen Waverley, Vic, 3150, Austalia

Computerised tomography (CT) colonography has been a rapidly evolving subject over the last few years due to a combination of clinical need and technological advances. The technique has moved from primarily a research tool to a commonly accepted examination in the clinical diagnostic armoury. Its position in the diagnostic hierarchy for colonic disorders is continuing to evolve.

Colorectal carcinoma is the second most common malignancy in the Western world and the third worldwide. Colorectal cancer is an optimal disease for screening with the majority of cases thought to arise from precancerous polyps that have a long latency time, in the order of 5–10 years before becoming malignant. Removal of the polyp removes the risk from that lesion. A more complete discussion is found in the foreword, by the eminent gastrointestinal radiologist Professor Richard M. Gore, which sets the tone for this publication.

Computerised tomography colonography currently performed in Australia is almost exclusively for diagnostic purposes and usually follows a failed optical colonoscopy. Worldwide trends and experience suggests this will change in future.

The Authors A/Professor Mangland and Professor Schima along with contributing editor, A/Professor Grasser, have a wide experience in this field with numerous publications in peer-reviewed journals.

As the title suggests, the aim of this book is to provide a practical guide to all aspects of CT colonography. This has been achieved by dividing the topic up into clearly defined logical chapters. These are: indications/contraindications, examination, image interpretation, findings, how to generate a useful report, screening and training. The layout with high-quality images, text boxes and highlighted key points makes for very easy reading and reference. This is a book that is easy to read for a quick 5 minutes or to sit down for a long educational session. The use of headings for each topic within a chapter makes for a quick reference guide without needing to plough through pages in order to obtain the required relevant information. A bibliography is provided at the end of each chapter, these are extensive and current in directing the reader to appropriate peer-reviewed literature. Throughout the book, reference is made to the European Society of Gastrointestinal and Abdominal Radiology (ESGAR) consensus statement on CT colonography produced in 2012. This is the latest consensus of leading European opinion leaders on quality standards in CT colonography.

The examination technique chapter sets out the range of different preparations, faecal tagging, antispasmodics, methods of colonic distension and scanning protocols. Each method is clearly discussed, with dot point protocols produced in ‘how to do it’ text boxes. The reader would be able to easily make a choice of techniques to suit their environment and patient populations. These techniques are comprehensively described with the advantages and disadvantages discussed where relevant.

For the reporting clinicians, the chapters on image interpretation and findings at CT colonoscopy are comprehensive and well set out in line with leading texts in this area. The practical guide element on how to produce a useful report, the use of C-RADS classification system and how to train in CT colonoscopy are innovative and instructive. The guide on how to generate a useful report is a useful tool for those clinicians already reporting CT colonography who wish to critically examine their own reporting techniques.

This book achieves its aims and much more being as authoritative on the topic as is practically possible. It is eminently suitable for radiographers and clinicians wishing to learn the technique of CT colonography.

For the clinician wishing to report CT colonography training is a prerequisite, with many countries requiring accreditation and ongoing competency. This book contains all the relevant information to serve as a standard training text. In addition, it is an easily referenced text for ongoing education. For those centres performing CT colonography this book could serve as the basis for a 360° review of the department's current practices with a view to ensuring the highest quality studies and reports are produced. This is particularly relevant in environments where limited numbers of examinations are performed.

